# Filter Characteristics Influencing Circulating Tumor Cell Enrichment from Whole Blood

**DOI:** 10.1371/journal.pone.0061770

**Published:** 2013-04-23

**Authors:** Frank A. W. Coumans, Guus van Dalum, Markus Beck, Leon W. M. M. Terstappen

**Affiliations:** Medical Cell BioPhysics, MIRA Institute, University of Twente, Enschede, The Netherlands; University of Arizona, United States of America

## Abstract

A variety of filters assays have been described to enrich circulating tumor cells (CTC) based on differences in physical characteristics of blood cells and CTC. In this study we evaluate different filter types to derive the properties of the ideal filter for CTC enrichment. Between 0.1 and 10 mL of whole blood spiked with cells from tumor cell lines were passed through silicon nitride microsieves, polymer track-etched filters and metal TEM grids with various pore sizes. The recovery and size of 9 different culture cell lines was determined and compared to the size of EpCAM+CK+CD45−DNA+ CTC from patients with metastatic breast, colorectal and prostate cancer. The 8 µm track-etched filter and the 5 µm microsieve had the best performance on MDA-231, PC3-9 and SKBR-3 cells, enriching >80% of cells from whole blood. TEM grids had poor recovery of ∼25%. Median diameter of cell lines ranged from 10.9–19.0 µm, compared to 13.1, 10.7, and 11.0 µm for breast, prostate and colorectal CTC, respectively. The 11.4 µm COLO-320 cell line had the lowest recovery of 17%. The ideal filter for CTC enrichment is constructed of a stiff, flat material, is inert to blood cells, has at least 100,000 regularly spaced 5 µm pores for 1 ml of blood with a ≤10% porosity. While cell size is an important factor in determining recovery, other factors must be involved as well. To evaluate a filtration procedure, cell lines with a median size of 11–13 µm should be used to challenge the system.

## Introduction

Circulating tumor cells (CTC) predict survival in patients with various metastatic cancers [1]–[8]. Enumeration of these CTC is a great technological challenge [9]. The lack of a positive control complicates assay development, as the number of CTC in patient samples is unknown. No detection of CTC in healthy controls and relatively high recovery of tumor cells spiked into blood samples are frequently used to gauge the performance of a given assay, yet without proof that the frequency of these cells relates to survival it remains uncertain whether “true” CTC are enumerated. CTC are extremely rare cells typically 1–10 CTC among ∼6×10^6^ leukocytes, ∼2×10^8^ platelets and ∼4×10^9^ erythrocytes per ml of blood [10]. This implies that any assay for their enumeration must be able to handle a large number of cells. Examination of a large number of immunofluorescently labeled cells increases the influence of non-specific binding which is inherent to immunofluorescence staining of cells. A non-specific background of 0.01% may be acceptable for routine immunophenotyping, but for rare event detection this would result in detection of 100 “CTC” after analysis of 1,000,000 leukocytes. To increase the specificity of CTC detection, the number of analyzed cells needs to be reduced. While some assays only perform an erythrocyte lysis or density separation [11]–[15] other assays achieve enhanced enrichment by leukocyte depletion [16], [17], size based filtration [18]–[24] or antibody based enrichment [25]–[31]. Each approach has its drawback. CTC could be lost due to the effect of erythrocyte lysis agents and CTC could be lost by employing a density separation as the range of densities of CTC is unknown. Size based filtration is antigen expression independent, but will miss smaller CTC and tumor micro particles, both of which are clinically relevant [32]. Antibody based methods are insensitive to variations in size, but don’t enrich tumor cells that have low expression of the target antigen. The epithelial cell adhesion molecule (EpCAM) is frequently used for CTC enrichment as it has little or no expression on leukocytes, and is expressed by the CTC in most patients [12], [32], [33].

Filtration was recently proposed for CTC enrichment [18]–[24] and previously we have reported on the filtration parameters important for enrichment of CTC from whole blood by filtration [34]. In this study we investigate the properties of the ideal filter for CTC recovery such as pore size, spacing between pores, number of pores, filter thickness and filter surface material. Filtration parameters such as dilution, fixation en flow rate are kept constant. In addition, the size distribution of CTC in metastatic breast, prostate and colorectal cancer and a variety of cell lines was determined to aid in choosing a cell line that can be used as an adequate model for optimization of filtration based CTC assays.

## Materials and Methods

### Blood Samples

Healthy volunteers aged 20–55 provided informed consent prior to donating blood. The study protocol was approved by the METC Twente ethics committee. Healthy was defined as no prior history of cancer or blood transmittable disease. Blood was drawn into EDTA vacutainers (BD, Franklin Lakes, NJ, USA) and processed within 8 hours after draw. Unless otherwise noted, each data point within one experiment represents the average of measurements on three different donors. Image archives from patients enrolled in studies with metastatic breast (IC 2006-04 [2], N = 247), colorectal (CAIRO-2 [3], N = 487) and prostate (IMMC-38 [4], N = 185) cancer patients were used for determination of CTC size.

### Cell Culture

Breast carcinoma cell lines SKBR-3, MDA-231, MDA-468 and MCF-7, prostate carcinoma cell line PC3-9, colorectal carcinoma cell lines COLO-320, SW-480, and hematopoietic cell lines HL-60, K-562 were used in various recovery experiments. All cell lines where obtained from ATCC (Manassa, VA, USA), except for the PC3-9 cell line, this sub-clone of the PC3 cell line [13] was kindly provided by Imunnicon (Huntingdon valley, PA, USA). PC3-9 were cultured using RPMI (Sigma, St. Louis, MO, USA) while the others were cultured in Dulbecco’s modified eagle medium (Sigma). Culture media were supplemented with 10% fetal calf serum (Gibco, Invitrogen, Carlsbad, CA, USA), 1% l-glutamin (Sigma) and 1% penicillin-streptomycin (Gibco). For MDA-468, the penicillin-streptomycin was replaced with 1% gentamicin and 1 mM pyruvate (Sigma). To eliminate issues with non-specific staining of blood cells or other debris on the filters evaluated, cells were stained before spiking into blood by incubation in culture media for 24 hours at 37°C with 50 µM CellTracker Green Bodipy and/or 5 µM CellTracker Orange CMTMR (both Invitrogen). Cells were harvested using 0.05% trypsin (Gibco) for 5 minutes at 37°C. The exact concentration of a cell suspension for spiking was determined using a flow cytometer (FACSAria II, BD) by placing the suspension in Trucount tubes (BD), staining with Hoechst 33342 (Invitrogen) and counting Hoechst and CellTracker positive events, with forward and side scatter signals typical for cells.

### Different Filter Types

**Figure 1:**
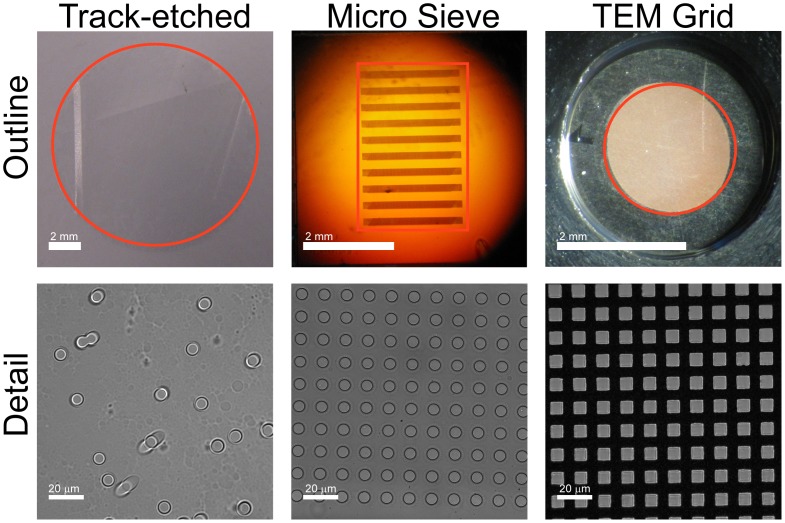
Overview of compared filters. The outline images show a photograph of the track-etch filter, the microsieve and the transmission electron microscopy (TEM) grid. The perforated area of each filter is indicated in red. The microsieve contains perforated horizontal bars alternated by support bars, giving rise to the horizontal pattern. The detail shows dark field images for the three filters. Spacing of the pores is random for the track-etch filters, leading to occasional double pores. Microsieves and TEM grids have periodical pore spacing.

Filters with diverse properties were selected for evaluation and characterized in figure 1 and table 1. This included polycarbonate track-etched filters with a pore diameter of 5, 8, and 10 µm (Whatman Nucleopore, GE, Kent, UK); silica nitride microsieves with a pore diameter of 5, 6, 7, 8, 9 and 10 µm (Aquamarijn, Zutphen, Netherlands) and copper and nickel TEM grids with 7.5 µm square pores (Gilder Grids, Grantham, UK). All filters except microsieves were mounted in 13 mm filter holders (Swinney, Pall, Mijdrecht, Netherlands). A ring was made to mount the TEM grids into the filter holders and microsieves were mounted in holders designed to hold the microsieves (TCO, Twente University, Enschede, Netherlands). Before use, a filter was placed in a holder, primed with PBS and placed in vacuum for 30 minutes. The vacuum was sufficient to make the PBS boil at room temperature thereby displacing any trapped air from the filter pores. At the end of this procedure the filters are still immersed in PBS.

**Table 1:** Properties of all tested filters.

Type	Material	Pore spacing	Thickness	Area	Pore size	N pores	Porosity
			um	mm∧2	um	×1000	%
Track-etched	PC	Random	10	102.1	5	350.9	6.7
				“	8	91.8	4.5
				“	10	88.2	6.8
Microsieve	Si_3_N_4_	Regular	1	0.6	5	0.16	0.5
				6.2	5	115.4	36.5
				2.7	5	25.9	18.8
				“	6	17.9	18.7
				“	7	15.2	21.7
				“	8	11.8	22.0
				“	9	9.4	22.1
				“	10	7.4	21.5
TEM grid	Copper	Regular	18	2.6	7½	17.4	36.0
	Nickel			“	“	“	“

PC: PolyCarbonate, Si_3_N_4_: Silicon Nitride, TEM: transmission electron microscopy, TEM grid pores are square, others are round.

### Setup

A setup for filtration of blood samples was constructed that allows for precise control of sample flow rate and dilution factor. The flow through the filter consists of two parts, the sample flow and a phosphate buffered saline (PBS) flow. If desired, the PBS flow can be used to dilute the sample. Dilution of 1:x means that 1 part sample is diluted in x-1 parts of PBS. For the sample flow, a sample is loaded into a 1 mL or 50 mL syringe (Plastipak, BD, Franklin Lakes, NJ, USA) with a 21 gauge needle (Microlance 3, BD) and placed onto a NE-1000 syringe pump (New Era Pump Systems, Farmingdale, NY, USA). The PBS flow originates from a stainless steel tank (Alloy products, Waukesha, WI, USA) pressurized with 2 bar N2. To reduce uptake of N2 into the PBS, the PBS is contained inside a plastic bladder inside a water filled tank. PBS is filtered by an in-line 0.2 µm filter (mini Kleenpak, Pall, Mijdrecht, The Netherlands) and the flow rate is controlled by a flow sensor (CoriFlow, Bronkhorst, Veenendaal, The Netherlands). The pressure difference across the filter is measured by a 0–300 mbar pressure sensor (PR-41X, Keller, Winterthur, Switzerland). By supplying a constant sample flow, the pressure across the filter increases until equilibrium is achieved between the number of cells that are pushed through the filter and the number of cells that arrive from the incoming sample flow. The setup stops filtering when the pressure exceeds 300 mbar to prevent damage to the setup. Before a sample is run the system is cleared from all air bubbles and a filter holder is attached. The holder is flushed with PBS at 1600 mL/h for 10 seconds, after which flow is stopped and the pressure measured is used as a bias. Unless otherwise specified the sample flow rate is then set at 25 mL/h and is diluted with 75 mL/h phosphate buffered saline (PBS) during injection. This sample flow rate resulted in a low pressure difference across the filter as previously shown [34].

### Detection of Recovered Cells

After sample injection stopped, the PBS flow was increased to 100 mL/h for 2 minutes to clear any residual blood from the filter holder. Next, the filter was fixed with an ethanol series increasing up to 100% and stained with 8 µM Hoechst 33342 in PBS for 10 minutes. The filter was mounted on a microscope slide with 75% glycerol. Digital images of the staining pattern in three fluorescence channels (DAPI, EGFP, R-PE filter sets, Chroma, Bellows Falls, VT, USA) were acquired with an Eclipse 400 epi-fluorescence microscope (Nikon, Tokyo, Japan) with CCD camera (C4742–95, Hamamatsu, Hamamatsu, Japan) and a 4x/NA0.13 objective or 10x/NA0.45 objective (both Nikon). The number of nucleated cells was enumerated automatically using an algorithm developed in Matlab 2009a (Mathworks, Natick, MA, USA) with DIPimage plugin (Delft Technical University, Delft, Netherlands). The number of cells from the tumor cell lines was determined using false color overlays of the different fluorescent stains, counting all objects of appropriate size and morphology, containing a nucleus, and having the correct color in the false color image. Recovery was defined as the percentage of spiked cells identified on the filter.

### Comparison of Different Versions of Each Filter Type

For comparison of the different filters, MDA-231, SKBR-3 and PC3-9 cells were spiked into whole blood at a concentration of 300 cells per mL blood for each cell type. MDA-231 cells were pre-stained with CellTracker orange, PC3-9 were pre-stained with CellTracker green, and SKBR-3 with both CellTracker orange and green. Cell recovery was determined as described above. The best version of each filter type was chosen for further experiments.

### Linearity of Recovery

To determine linearity of recovery pre-stained MDA-231 cells were spiked into 1 mL of blood at concentrations of 2, 10, 100, 1,000, 10,000, 30,000 (microsieve only), 100,000 (track-etched only) cells. The maximum spike was similar to the number of pores for each filter. MDA-231 cell concentration was determined on the flow cytometer, except for the spikes of (nominally) 2 and 10 cells, where the exact number spiked cells was determined by microscope inspection of each spike drop. To limit the total volume of blood needed for the experiments 1 mL of blood was aspirated into a syringe and 10 µL of culture cell suspension was pipetted into the front of the syringe. The syringe was inverted and left for a few minutes to allow cells to disperse before the sample was used for filtering. Downside of this procedure is that cells are more likely to arrive early on in the filtration process, leading to slightly lower recoveries than when the cells were evenly distributed throughout the blood sample before loading the syringe.

### Impact of Different Sample Volumes

The impact of cell enrichment from different volumes of whole blood was tested by spiking 300 MDA-231 cells into blood volumes of 0.1, 1 and 10 mL. The recovery of spiked cells was determined by counting MDA-231 cells on the filters. Additional experiments were conducted to determine recovery, while maintaining flow rates per pore that are comparable between track-etched filters and microsieves. The number of pores in a track-etched filter is approximately 10 times higher than the number of pores in a microsieve. Since the flow of cells per pore makes a large difference in pressure across the filter, and thus recovery, the recovery after spiking 30 MDA-231 cells into 0.1 mL of whole blood, when filtered at a total flow rate of 10 mL/h was determined. The flow per pore is then equivalent to flow per track-etched pore when spiking 300 MDA-231 cells into 1 mL of whole blood at a total flow rate of 100 mL/h.

### Cell Size Determination

The size of the cells was determined by the Coulter principle and by an image analysis algorithm. A Coulter counter pipette (Scepter, Millipore, Billerica, MA, USA) was used to determine the size of all cell lines and leukocytes. Leukocytes were obtained from the buffy coat of 10 mL whole blood centrifuged at 300×g for 10 minutes. Size of cells was determined by imaging using an algorithm developed for automated counting of CTC [33]. This algorithm automatically identifies CK+DNA+CD45− CTC and can output the size of the area occupied by the CTC. Size of CTC from patients with metastatic breast cancer [2], colorectal cancer [3], and prostate cancer [4] was determined using image archives from these studies. For tumor cell lines we used the same CTC algorithm and for detection of leukocytes the algorithm was modified such that CK−DNA+CD45+ objects were counted. Because cells flatten when pulled against the glass slide inside the sample cartridge, the surface area of a cell cannot be used directly to derive the diameter of the cell in suspension. Instead we assumed cells to be cylindrical in shape with a volume of the imaging area *A* times a constant thickness *D*:
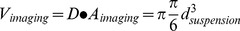
(1)


To find the diameter of a CTC in suspension from imaging, *D* was least squares fit to size data of SKBR-3, PC3-9, MDA-231 culture cells and leukocytes [35], which were determined using both Coulter principle and imaging.

### Recovery for Different Cell Lines

To determine the relation between the size of cells from the tumor cell lines and recovery, 300 cells were spiked into 1 mL of blood and the spiked samples were filtered through the best filter for each filter type. Spiking was achieved as described in “Linearity of recovery”. Tumor cell lines included breast cancer cell lines SKBR-3, MDA-231, MDA-468 and MCF-7, prostate cancer cell line PC3-9, colorectal cancer cell lines COLO-320, SW-480, and hematopoietic cell lines HL-60, K-562. For each cell line the recovery was determined in duplo.

### Staining of Leukocytes

One mL of unspiked whole blood was filtered through 8 µm track-etched filters to determine whether there is a subpopulation of leukocytes that is preferentially enriched. To preserve antigens, the filters were fixed in 0.4% PFA (paraformaldehyde) for 10 minutes instead of the ethanol fixation used in other experiments. The filters were then stained for 15 minutes at room temperature with a staining cocktail containing nuclear dye Hoechst 33342 (8 µM, Invitrogen), and antibody conjugates CD13-PE (BD), CD14-APC (EXBIO, Prague, Czech), CD20-FITC (BioLegend, San Diego, CA, USA), CD4-FITC (MACS, Cologne, Germany), CD8-FITC (BD) at concentrations per the manufacturers’ recommendations. The filters were imaged on a fluorescent microscope, followed by cell enumeration. At least 100 nucleated cells were counted and differentiated between granulocytes (CD13+, CD14−), monocytes (CD13+, CD14+), lymphocytes (CD20+ or CD4+ or CD8+, all CD14−) and others (negative for all). As a reference 1 mL of whole blood was stained, and leukocyte types were enumerated in a smear of this sample.

## Results

### Cell Enumeration is Easiest on a Stiff Filter with Low Porosity

**Figure 2:**
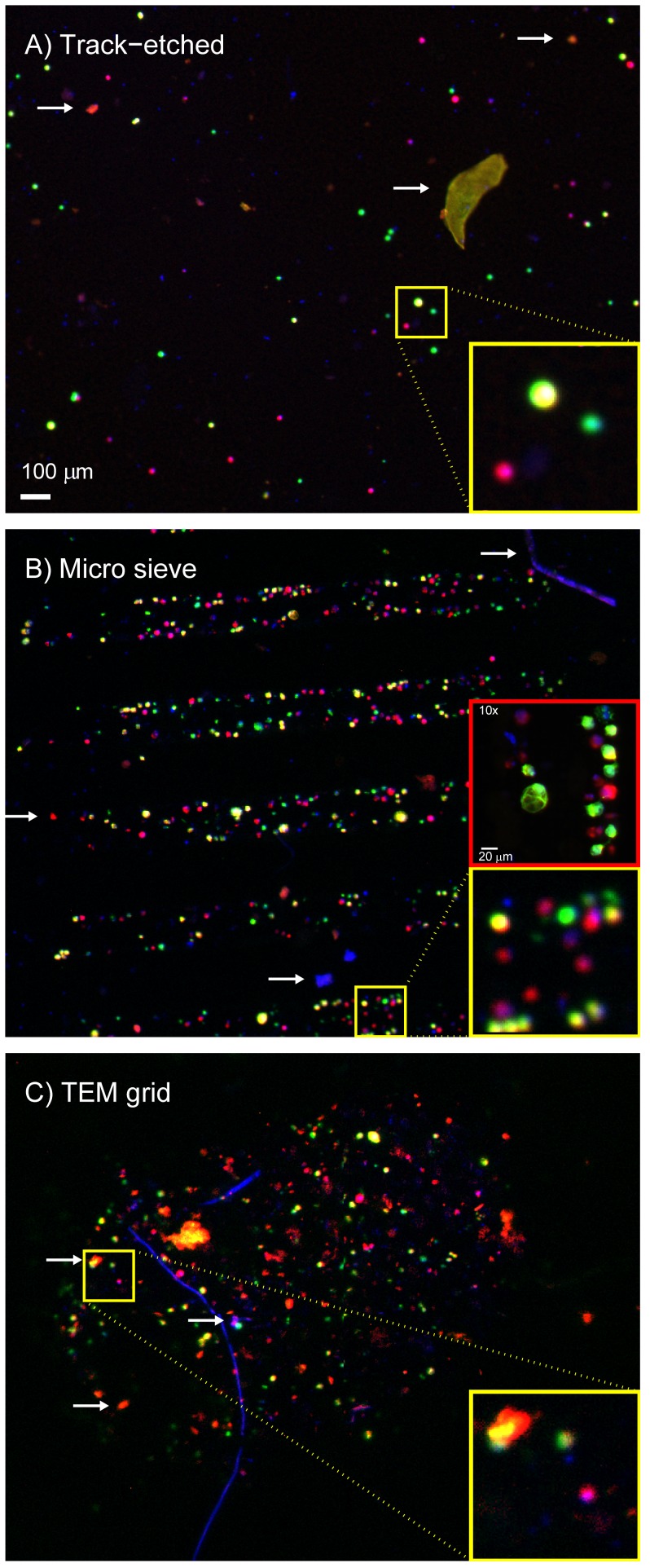
Images of cells on all filters. Panel A shows a track-etched filter, panel B a microsieve filter and panel C a TEM grid filter. The images show false color fluorescent images taken of pre-stained cells enriched from a whole blood sample. Hoechst 33342 stain is shown in blue, Celltracker Orange in red, and Celltracker Green in green. In the false color MDA-231 cells are red, PC3-9 cells green and SKBR-3 cells yellow. Several types of autofluorescent and/or Hoechst positive debris were found on the filters, examples are indicated with white arrows. This debris is also found in unspiked samples. Due to the low number of pores and the high porosity of the microsieves and TEM grids, cells are very close to each other. To better distinguish cells on these filters, imaging with a 10x objective was necessary for determination of cell recovery as shown in the red insert in panel B. This was not possible with the track-etched filters because they were not sufficiently flat to have all cells in focus.

Images of the filters after filtration of a spiked 1 mL blood sample are shown in figure 2. Images were taken with a 4x/NA0.13 objective. The track-etched filter deformed during filtration and as a result could not be imaged with a 10x/NA0.45 objective; 5–15% of the filter was too far out of focus to allow enumeration of positive cells in this area. The high porosity and low number of pores of the microsieves and the TEM grids resulted in a cell density too high to reliably distinguish adjacent cells when imaged with a 4x/NA0.13 objective, see figure 2 panels B and C, therefore these filters were imaged with the 10x/NA0.45 objective for all experiments. A large part of the TEM grid is covered with a red blood cell layer, the image in panel C of figure 2 shows the only part of the filter that was not covered with red blood cells, comprising 17% of the total filter area. The ideal filter should maintain its planar form during filtration, it should not react with the sample and pores should be sufficiently separated to facilitate discrimination of cells.

### Increasing Pore Size Leads to Lower Recovery and Higher Sample Purity

**Figure 3:**
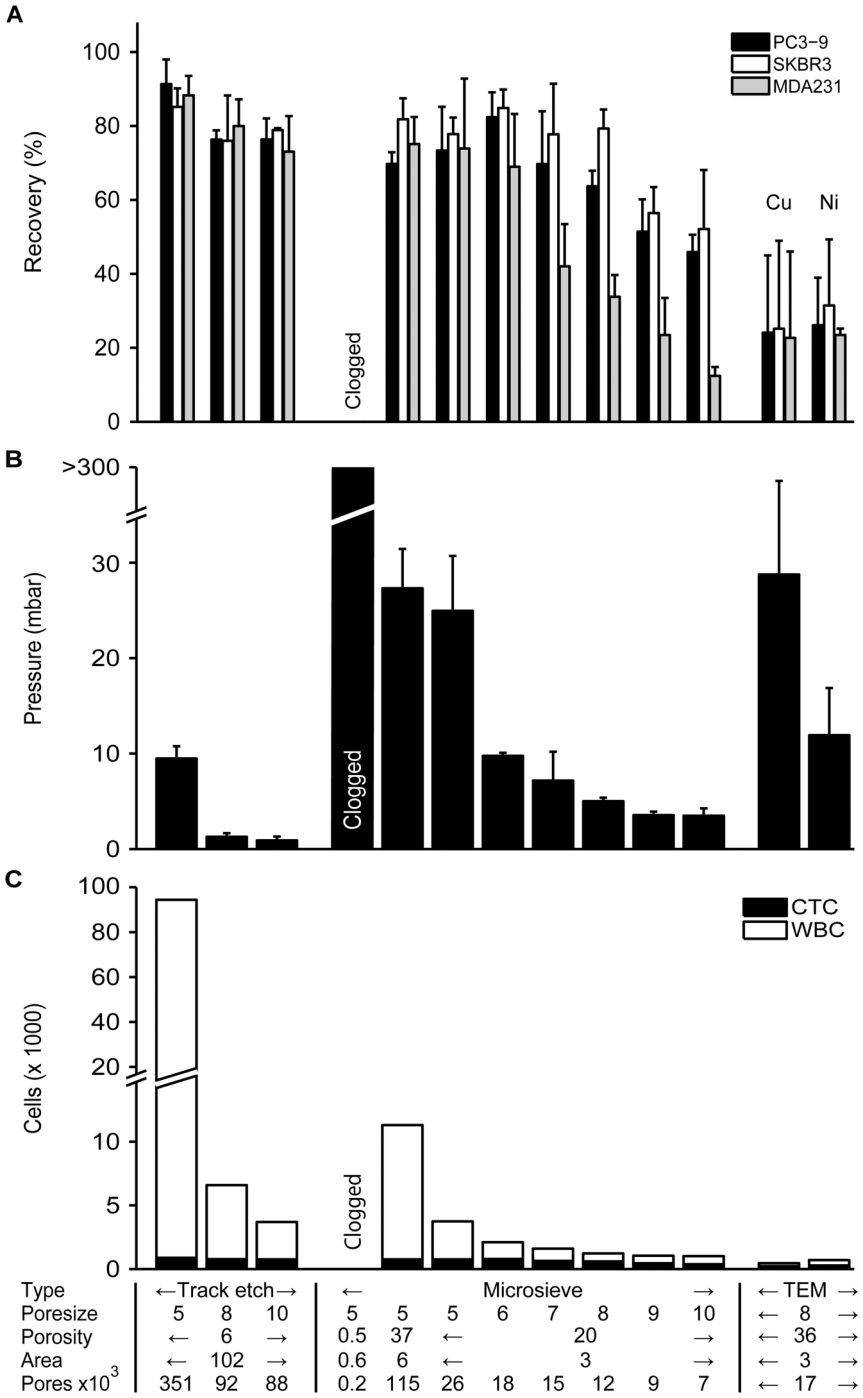
Comparison between different filter types. **Panel A** shows recovery for SKBR-3, PC3-9 and MDA-231 cell lines. Bar height represents recovery with whiskers showing 1 standard deviation. No recovery was determined for the 0.5% porosity microsieve due to clogging while filtering. **Panel B** shows the peak pressure achieved during filtration. Whiskers show 1 standard deviation. **Panel C** shows the number of nucleated cells on the filter, with the CTC fraction also shown. The X-axis summarizes the major differences between the filters and applies to all panels.

The results of the filter comparison are shown in figure 3. In panel A the % recovery is shown per cell type and per filter type. In panel B the pressure across the filter is shown and the sample purity is shown in panel C. The key filter properties are found at the bottom of panel C. Recovery of spiked cells is highest for filters with small pores. When the pore size is increased beyond 6 µm, recovery starts to reduce for MDA-231 cells, but this reduction seems be slower and to occur at a different size for the other two cell lines, at pores sizes of 7 µm for PC3-9 cells and 8 µm for SKBR-3 cells. The pressure across the filter was lowest for filters with larger pores and filters with more pores. 80–90% of the TEM grid filter was covered with a coat of red blood cells, only a small aperture in the middle remained open. Only 25% of spiked cells were recovered, all found in this aperture, however it is possible that more cells were underneath the red blood cell coat. Recovery was also reduced by the high pressure across the 8 µm TEM grids, which was much higher than the pressure across the 8 µm microsieves, while they have similar numbers of pores. Higher pressure also leads to lower recovery when comparing microsieves to track etched filters. The pressure across the microsieves of similar pore size to the track-etched filters was always higher and recovery was always lower. The 5 µm microsieve with 160 pores needed a pressure in excess of 300 mbar to handle the sample flow, which this setup cannot provide.

As long as there are sufficient pores to pass the sample, recovery seems to be insensitive to the number of pores. There is almost no difference between the microsieves with 115,000 pores and the one with 26,000 pores in terms of spiked cell recovery or pressure across the filter, but the number of leukocytes retained is 3.1 fold higher on the filter with 4.4 fold more pores. The number of leukocytes retained is the highest on the 5 µm track-etched filter. Less than 6% of pores are occupied by leukocytes for 8–10 µm track-etched filters and ≥6 µm microsieves.

The filters with highest yield and lowest leukocyte contamination were the 8 µm track-etched filter and the 5 µm microsieve with 26,000 pores, because they recovered >70% of the spiked cells, while retaining a low percentage of leukocytes. These two filters were selected for further comparison in the next experiments. The TEM grids had unsatisfactory recovery, and were not used in further experiments.

### Recovery is Constant Until Approximately 2% of Pores are Occupied

**Figure 4:**
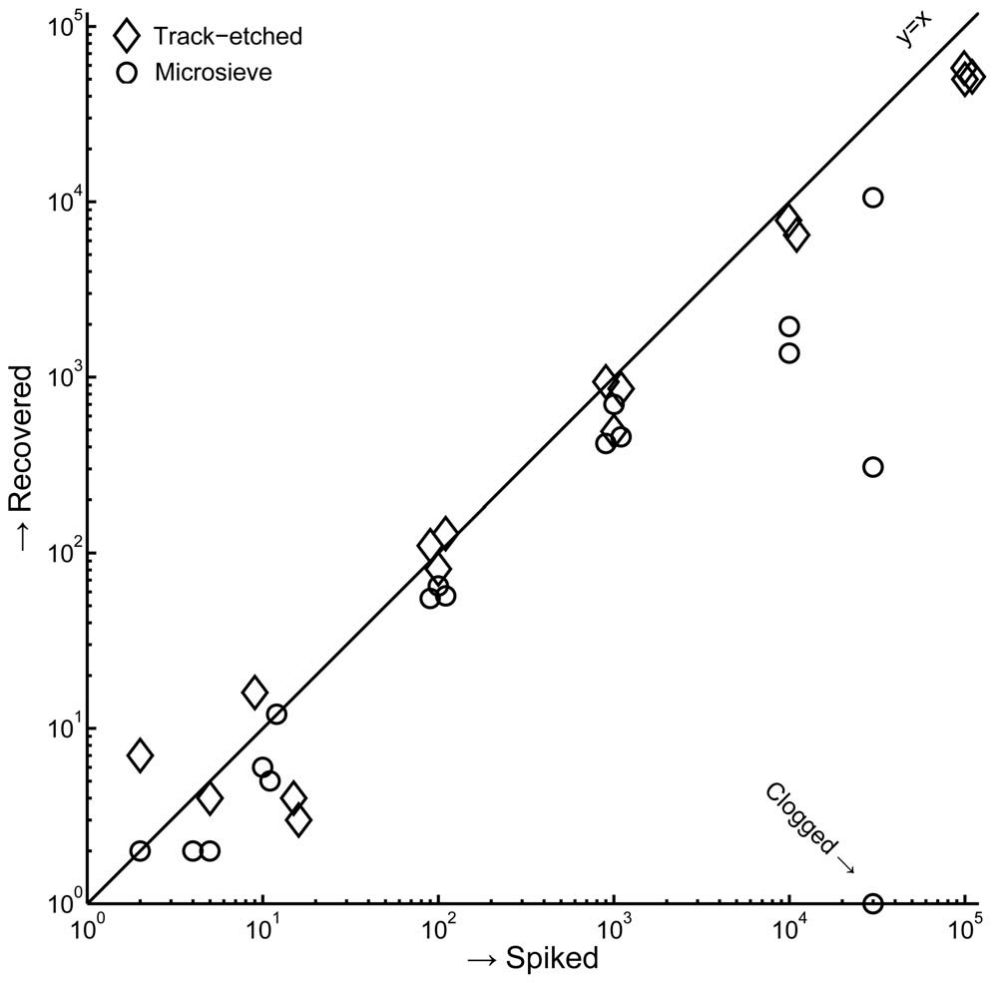
Linearity of recovery for 8 µm track-etched filters and 5 µm microsieves. Whole blood was spiked with 2, 10, 100, 1000, 10000, 30000 (microsieve only) or 100,000 (track-etch only) MDA-231. For spikes with target values of 2 and 10 cells, we determined the actual number of cells by microscope inspection of the cell drop prior to spiking. The counted spikes are shown, not the targets. Spikes of 10^4^ cells and higher were counted automatically. Spikes of 100 and higher were offset by up to 10% to enhance readability of the graph.

The number of MDA-231 cells recovered versus the number spiked cells is shown in figure 4. Recovery is constant for spikes of 0–100 cells on the microsieves (58% recovery, R^2^ = 0.98), and for spikes of 0–10,000 cells on the track-etched filters (67% recovery, R^2^ = 0.98). When a sufficient fraction of pores on the filter are blocked, the pressure during filtration is increased and recovery reduced [34]. Each cell occupies 3–5 of the 26,000 pores on the microsieves, while on the track-etched filters most cells occupy 1 of the 351,000 pores. Including all data in the linear fit reduces recovery and correlation both for the microsieves (12% recovery, R^2^ = 0.49) and for the track-etched filters (51% recovery, R^2^ = 0.91). One of the microsieves clogged at a spike of 30,000 cells, and average recovery for this spike was 10%. 2–5 false positives cells were counted on the track-etched filters, while none were counted on the microsieves.

### The Volume that can be Filtered is Limited by the Contaminant Concentration

**Figure 5:**
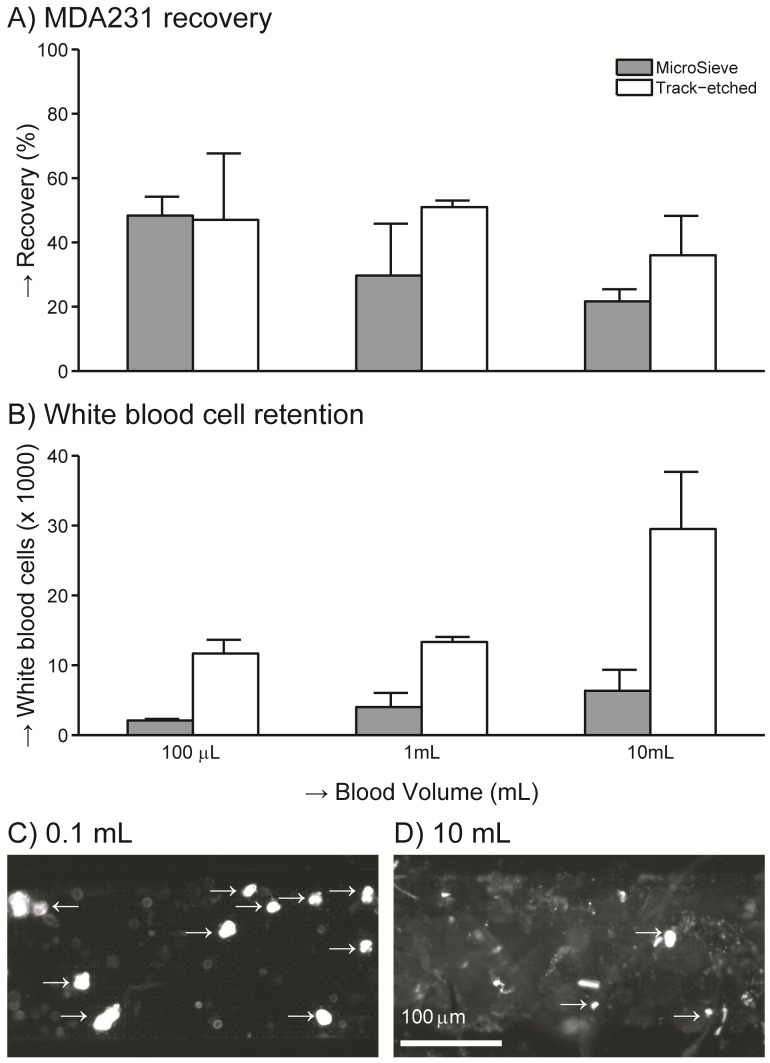
Impact of total blood volume. Recovery of 300 MDA-231 cells spiked into 0.1, 1 and 10 mL of whole blood determined on 5 µm microsieves and 8 µm track-etched filters. **Panel A** shows the % recovery of MDA-231 cells, **panel B** the number of white cells found on the filter. The images in **panel C and D** show the CellTracker Orange signal after microsieve filtration of 0.1 and 10 mL of whole spiked blood respectively. The saturated dots indicated with the arrows are counted as MDA-231 cells, the weaker fluorescence is autofluorescent material.

The recovery after spiking 300 MDA-231 cells into 0.1, 1 or 10 mL of whole blood is shown in figure 5, panel A. On track-etched filters recovery was 47%, 51%, 36% with volumes of 0.1, 1 and 10 mL, respectively. On microsieves recovery was 48%, 30%, 22% with volumes of 0.1, 1 and 10 mL, respectively. The amount of the debris, as indicated with white arrows in figure 2, as well as the number of leukocytes retained on the filter increased with increasing sample volume, see figure 5, panel B. This accumulation of debris leads to a high autofluorescent background as can be seen in panel D of figure 5, compared to panel C of figure 5. In addition an experiment was conducted where 30 MDA-231 cells were spiked into 0.1 mL of whole blood and filtered through a microsieve at 10 mL/h. This makes the flow per pore and the number of cells per pore equivalent to a spike of 300 MDA-231 cells in 1 mL of whole blood when filtered through a track-etched filter. Recovery under this condition was 57±31%.

### Monocytes are Retained More than Other Leukocytes

The selective enrichment of the leukocyte subpopulations on the track-etched filter is shown in table 2, the relative proportion of monocytes is increased by 165% at the cost of both granulocytes and lymphocytes. Of the 5·10^6^ leukocytes in a 1 mL sample 0.1% was recovered on the filter.

**Table 2:** Filtration retains slightly more monocytes than other white blood cells.

% of total	granulocytes	lymphocytes	monocytes	other
whole blood	65±4	29±5	5±5	1±0
on filter	58±3	27±3	14±2	1±0
% change	−11±3	−5±6	165±37	1±30

Numbers give % of total ± standard deviation determined on 5 healthy donors. Each data-point was determined by counting at least 300 cells.

### EpCAM+CK+CD45− CTC are Smaller than Typical Cells Derived from Tumor Cell Lines

**Figure 6:**
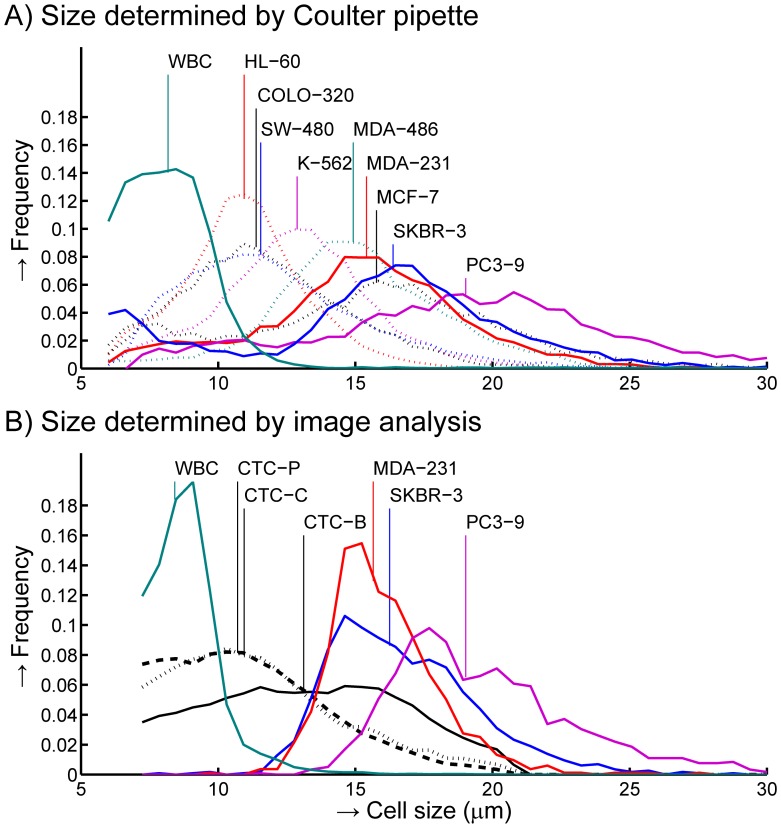
Size distribution of blood cells and various cancer cell lines. Blood cells include white and red blood cells and the hematopoetic cell lines HL-60 and K-562. Cancer cell lines include prostate cancer cell lines PC3-9, colorectal cancer cell lines COLO-320 and SW-480, breast cancer cell lines SKBR-3, MCF7, MDA-231 and MDA-468. Distributions are normalized to unit area. Vertical lines indicate the median size for each cell type. **Panel A** shows the size distribution as determined using a Coulter pipette. **Panel B** shows the size distribution as determined using image analysis for MDA-231, SKBR-3, PC3-9, leukocytes (WBC) and CTC from patients with metastatic breast (CTC-B, solid line), colorectal (CTC-C, dotted line) or prostate (CTC-P, dashed line) cancer.

A Coulter pipette was used to determine the size distributions of 9 tumor cell lines and leukocytes, shown in figure 6, panel A. The median size of leukocytes was 8.1 µm while cells from the tumor cell lines ranged from 10.9 to 19.0 µm. SKBR-3 shed small vesicles in the culture medium, which leads to a peak in the distribution near 6 µm, comprising up to 15% of total cells. The tumor cell line standard deviation relative to the median size (% CV) ranged between 19% (HL-60, MDA-468) to 31% (MCF-7). The size of MDA-231, SKBR-3 and PC3-9 cell lines was also determined using morphology parameters from an automated CTC counting algorithm, which automatically identifies EpCAM+CK+DNA+CD45− CTC, see figure 6 panel B. The cell thickness when imaging was found to be 5.2 µm by fitting the median diameter of these cell lines to the median diameter from the Coulter determination using equation 1. With this thickness, deviation between median size by imaging versus Coulter of SKBR-3, MDA-231, PC3-9 and leukocytes was less than 0.4 µm, The % CV of the distributions for imaging/Coulter was 16/26% for PC3-9, 15/30% for SKBR-3, 11/22% for MDA-231 and 16/77% for leukocytes. Applying equation 1 the median diameter of CTC was determined to be 10.7 µm for prostate, 11.0 µm for colorectal and 13.1 µm for breast CTC.

### Cell Lines with Size of CTC Typically have Low Recovery

**Figure 7:**
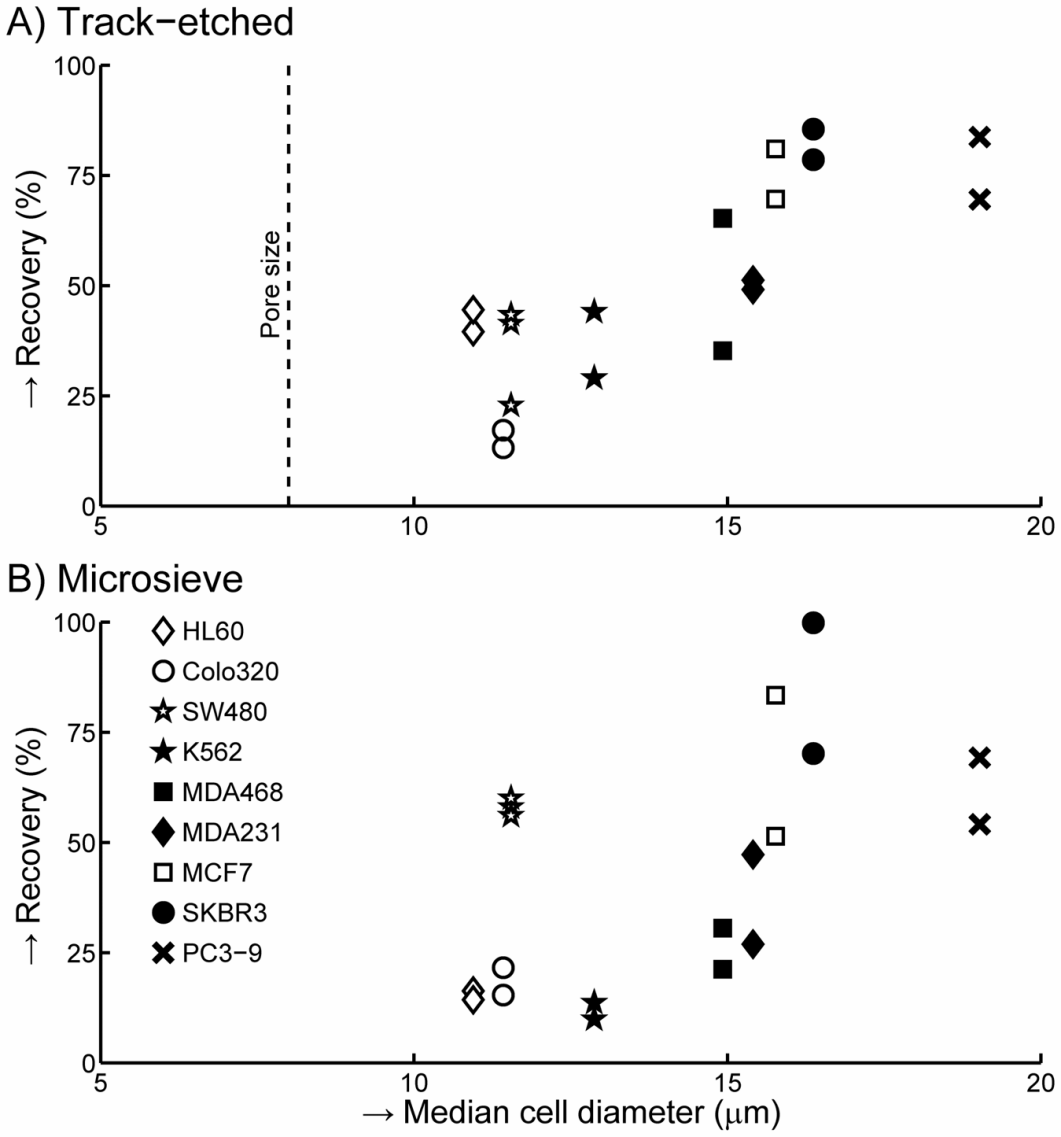
Recovery of different cell lines. 300 cells of each cell line were spiked into 1 mL of whole blood. Recovery obtained with 8 µm track-etched filters is shown in **panel A**, recovery with 5 µm microsieves is shown in **panel B**.

Three-hundred cells from 9 different tumor cell lines were spiked into 1 mL of blood and filtered through 8 µm track-etched filters and 5 µm microsieves. Recovery for duplicates of each experiment are shown in figure 7, panel A for track-etched filters and panel B for microsieves. The trend for both filter types shows that recovery increases with cell size, however different cell lines of similar size can have two-fold difference in recovery. A larger cell typically has higher recovery, but this is a weak trend, with large variations in recovery between cells of similar size. Average recovery of cells with sizes (10.8–12.9 µm) on the order of patients CTC (10.7–13.1 µm) was 33% (range 13–44%) on the track-etched filter and 29% (range 13–60%) on the microsieve with Colo-320, SW-480, HL-60 and K-562 cells. The high recovery of SW-480 cells on the microsieves was confirmed by measuring an additional time on both filter types. The third repeat matched the first two, and is thus not due to experimental error. A smaller track-etched pore size may improve recovery. We determined recovery for 5 µm pores by spiking Colo-320 or SW-480 cells into 1 mL of blood. Filtering a spiked sample with Colo-320 increased recovery from 15.2±2.8% (8 µm track-etched) to 19.6±7.5% (5 µm track-etched). With the SW-480 cell line, recovery increased from 35.9±11.3% to 70.8±10.4%.

## Discussion

We determined the size of CTC in suspension by comparing size determined by the Coulter principle to size determined by imaging. For this comparison, calibration of size by imaging versus size by the Coulter principle is needed. Good agreement on the median size was found, however the % CV on the size was larger by the Coulter principle than by the imaging approach, up to double for the cells from the tumor cell lines and almost five times higher for leukocytes. Possible explanations for these differences: - The imaging approach requires presence of a nucleus, while the Coulter approach determines size on all detected objects, including vesicles produced during culture of some cell lines, increasing CV. - EpCAM-magnetic enrichment and automatic detection of CK staining may favor enrichment and subsequent detection of larger objects, reducing CV with the imaging approach. - Leukocytes are a mixture of three main cell types of different sizes. Relatively high non-specific adhesion of granulocytes means that EpCAM enrichment captures granulocytes relative to other leukocytes and thus the size by imaging represents the size of granulocytes, resulting in a CV that is much lower than a true white blood cell mixture. Median size of EpCAM+CK+DNA+CD45− CTC found by the CellSearch system in metastatic breast is 13.1 µm, prostate 10.7 µm and colorectal cancer is 11.0 µm. This is much smaller than the size of most cells derived from tumor cell lines, figure 6. Assuming filtration recovers 100% of all cells in excess of a minimum size, table 3 shows an estimate of recovery for minimum size of 8, 10, 12, 14 and 16 µm derived from figure 6, panel B. For recoveries of 70% or higher, size based filtration methods need to recover CTC that are larger than 9.2 for prostate, 9.4 for colorectal and 10.8 µm for breast cancer. Unfortunately recovery with such small cells was poor, figure 7, and the 8 µm track-etched filter will probably not recover cells of 9.2 µm. Validation of a filtration assay using SKBR-3, MCF-7, PC3-9 or similarly sized cells may not have any relevance to CTC enrichment by means of filtration.

**Table 3:** Percentage of EpCAM+CK+CD45− CTC that can be recovered for various size cutoffs.

Size (µm)	Breast	Colorectal	Prostate
8	92	87	84
10	77	61	58
12	60	36	34
14	41	19	16
16	23	9	7

MDA-231 recovery determined during the filter comparison experiment was typically 10–20% higher than the recovery determined using subsequent experiments. This may be due to an error in the spike determination or due to misclassification of some SKBR-3 cells with weak CellTracker Green stain as MDA-231 cells in the comparison experiment or due to the spiking method used in all subsequent experiments. As a result of this method the spiked cells are captured on the filter early in the experiment and have more time to pass through the filter.

In the filter comparison shown in figure 3, we chose the 8 µm track-etched filters and the 5 µm microsieve as the best filters. We aimed for recovery >70% and the lowest leukocyte contamination possible. The rationale for these targets was that while high CTC recovery is desirable, low leukocyte recovery is also desirable, as they may stain slightly positive for EpCAM or cytokeratin stains. This complicates review and leads to false positives [36]. In addition, leukocytes complicate CTC analysis using techniques such as PCR [37]. However, average recovery was only 33% for cells with a size comparable to CTC. With such recovery high CTC recovery takes precedence over low leukocyte contamination. Changing from 8 to 5 µm track-etched filters resulted in acceptable recovery of 71% for SW-480 cells, but a still unacceptable 20% for COLO-320 cells. It is unknown which cell line is more similar to CTC.

While there was an association between recovery and cell size, see figure 7, it was not sufficient to explain the differences in recovery between the different cell lines. Additional factors contributing to recovery may be the deformability of cells [38], [39] or size of the nucleus [40]. It was surprising that the 5 µm microsieve performed similar to the 8 µm track-etched filter with matched flow per pore conditions. When passing through a 5 µm pore filter, the leukocyte membrane needs to deform 15% for a 1 µm thick filter and 30% for a 10 µm thick filter. As a result, a cell could be squeezed through the 1 µm thick microsieves more easily than through the 10 µm thick track-etched filters, which is countered by the smaller pore size. Ultimately we obtained the best result with a 5 µm pore, 10 µm thick track etched filters.

With the leukocytes we expected to see an enrichment of the larger monocytes (8.9–9.6 µm [41]) and depletion of the smaller lymphocytes (6.8–7.7 µm [41]). While table 2 shows monocytes were indeed enriched, the ratio between lymphocytes and granulocytes (8.0–9.1 µm [41]) was unchanged. We further expected hematopoietic cell lines to be more flexible than the epithelial cell lines, and thus to have much lower recovery. Surprisingly, recovery of hematopoietic HL-60 and K-562 cells is comparable to similarly sized epithelial SW-480 and COLO-320 cells.

The number of pores needed depends on the number of cells that need to be recovered (figure 4), and the number of cells that need to pass the filter (figure 5). Recovery on microsieves was lower than recovery on track-etched filters in most experiments, but using an 8 µm track-etched filter and a 5 µm microsieve with comparable numbers of cells per pore, recovery of MDA-231 cells was 51% and 57% respectively. A further factor in determining the number of pores needed is the contamination with debris as seen in figure 2. A larger whole blood volume contains more debris, which may block multiple pores. For blood samples of 1 mL, 100.000 pores are adequate. To prevent reduced recovery the number of CTC needs to be less than 2000 and each cell should block one pore only. The number of metastatic patients with more than 2000 CTC/mL is less than 2% [42], reduced recovery for these patients would not affect the ability to perform phenotyping of the individual CTC in these patients since there would still be many CTC left.

The number of pores blocked per captured cell relates to the porosity of the filter. The 5 µm microsieves had high porosity of 20%, blocking 4–6 pores per captured MDA-231 cell, while with the 5% porosity 8 µm track-etched filter less than 10% of MDA-231 cells blocked more than one pore. These were pores that were close to each other due to random pore location on the track-etched filter. On high porosity filters distinguishing two adjacent cells is complicated by poor separation. This made enumeration of cells on the microsieves more difficult than on the track-etched filter. A porosity of up to 10% on a filter with regularly spaced pores allows for discrimination of cells on adjacent pores, and each cell would block a single pore.

The material of the filter must provide at least two properties. First the material must be compatible with blood. Interaction of erythrocytes with the filter surface resulted in low recovery on the TEM grids. Further, it must allow for imaging of CTC at sufficient resolution to discriminate between false positive leukocytes and true CTC. With the CellSearch platform, a 10X/NA0.45 objective is adequate for this purpose, but we could not image the track-etched filter at this resolution because it had deformed during filtration. The filter material must be sufficiently stiff to prevent deformation during filtration. Imaging at 4x/NA0.13 of the track-etched filters would have been a problem for adequate identification if we had used EpCAM/CK immuno-fluorescence for identification of culture cells, rather than using CellTracker pre-stained cells. While solutions for this problem exist [19], they are time consuming and can be complicated. A third property that is desirable is a CTC specific coating of the filter surface, for example with EpCAM. Such a coating may increase recovery of CTC, while retention of leukocytes is unaffected. Whether a hydrophilic or hydrophobic filter surface has better properties for enrichment of CTC by means of filtration is also of interest. This could be determined by coating microsieves with hydrophilic coatings [43].

In summary, the ideal filter for CTC enrichment from 10 ml of whole blood has a pore size of about 5 µm, thickness of at least 10 µm, at least 100,000 regularly spaced pores, a porosity of 10% or less and is constructed of a stiff, flat material, which does not interact with blood cells. While cell size is an important factor in determining recovery, other factors must be involved in determining whether a cell can pass as well. To evaluate a filtration procedure, cell lines with a median size of 11–13 µm should be used to challenge the system, such as Colo-320, SW-480 and not cell lines significantly larger than CTC.

## References

[1] Cristofanilli M, Budd GT, Ellis MJ, Stopeck A, Matera J, et al (2004) Circulating tumor cells, disease progression, and survival in metastatic breast cancer. N Engl J Med 351: 781–791. 15317891 10.1056/NEJMoa040766

[2] Pierga JY, Hajage D, Bachelot T, Delaloge S, Brain E, et al (2012) High independent prognostic and predictive value of circulating tumor cells compared with serum tumor markers in a large prospective trial in first-line chemotherapy for metastatic breast cancer patients. Ann Oncol 23: 618–24. 21642515 10.1093/annonc/mdr263

[3] Tol J, Koopman M, Miller MC, Tibbe A, Cats A, et al (2010) Circulating tumour cells early predict progression-free and overall survival in advanced colorectal cancer patients treated with chemotherapy and targeted agents. Ann Oncol 21: 1006–1012. 19861577 10.1093/annonc/mdp463

[4] de Bono JS, Scher HI, Montgomery RB, Parker C, Miller MC, et al (2008) Circulating Tumor Cells Predict Survival Benefit from Treatment in Metastatic Castration-Resistant Prostate Cancer. Clin Cancer Res 14: 6302–6309. 18829513 10.1158/1078-0432.CCR-08-0872

[5] Rao C, Bui T, Connelly M, Doyle G, Karydis I, et al (2011) Circulating melanoma cells and survival in metastatic melanoma. Int J Oncol 38: 755–760. 21206975 10.3892/ijo.2011.896

[6] Matsusaka S, Chin K, Ogura M, Suenaga M, Shinozaki E, et al (2010) Circulating tumor cells as a surrogate marker for determining response to chemotherapy in patients with advanced gastric cancer. Cancer Sci 101: 1067–1071. 20219073 10.1111/j.1349-7006.2010.01492.x PMC11159155

[7] Krebs MG, Sloane R, Priest L, Lancashire L, Hou JM, et al (2011) Evaluation and prognostic significance of circulating tumor cells in patients with non-small-cell lung cancer. J Clin Oncol 29: 1556–1563. 21422424 10.1200/JCO.2010.28.7045

[8] Hiltermann TJN, Liesker JJW, van den Berg A, Schouwink JH, Wijnands WJA, et al (2012) Circulating tumor cells in small cell lung cancer, a predictive and prognostic factor. Ann Oncol 23: 2937–2942. 22689177 10.1093/annonc/mds138

[9] den Toonder J (2011) Circulating tumor cells: the Grand Challenge. Lab Chip 11: 375–377. 21206959 10.1039/c0lc90100h

[10] Allard WJ, Matera J, Miller MC, Repollet M, Connelly MC, et al (2004) Tumor cells circulate in the peripheral blood of all major carcinomas but not in healthy subjects or patients with nonmalignant diseases. Clin Cancer Res 10: 6897–6904. 15501967 10.1158/1078-0432.CCR-04-0378

[11] Hsieh HB, Marrinucci D, Bethel K, Curry DN, Humphrey M, et al (2006) High speed detection of circulating tumor cells. Biosens Bioelectron 21: 1893–1899. 16464570 10.1016/j.bios.2005.12.024

[12] Pachmann K, Camara O, Kavallaris A, Krauspe S, Malarski N, et al (2008) Monitoring the response of circulating epithelial tumor cells to adjuvant chemotherapy in breast cancer allows detection of patients at risk of early relapse. J Clin Oncol 26: 1208–1215. 18323545 10.1200/JCO.2007.13.6523

[13] Rao CG, Chianese D, Doyle GV, Miller MC, Russell T, et al (2005) Expression of epithelial cell adhesion molecule in carcinoma cells present in blood and primary and metastatic tumors. Int J Oncol 27: 49–57. 15942643

[14] Haubert T, Contini V, Grimes S, Jones R, Wardlaw SC (2008) Buffy coat separator float system and method. US Patent 8,114,680 B2.

[15] Rosenberg R, Gertler R, Friederichs J, Fuehrer K, Dahm M, et al (2002) Comparison of two density gradient centrifugation systems for the enrichment of disseminated tumor cells in blood. Cytometry 49: 150–158. 12454978 10.1002/cyto.10161

[16] Balasubramanian P, Yang L, Lang JC, Jatana KR, Schuller D, et al (2009) Confocal images of circulating tumor cells obtained using a methodology and technology that removes normal cells. Mol Pharm 6: 1402–1408. 19445481 10.1021/mp9000519 PMC2829323

[17] Alix-Panabieres C, Rebillard X, Brouillet JP, Barbotte E, Iborra F, et al (2005) Detection of circulating prostate-specific antigen-secreting cells in prostate cancer patients. Clin Chem 51: 1538–1541. 16040853 10.1373/clinchem.2005.049445

[18] Hofman VJ, Ilie MI, Bonnetaud C, Selva E, Long E, et al (2010) Cytopathologic Detection of Circulating Tumor Cells Using the Isolation by Size of Epithelial Tumor Cell Method: Promises and Pitfalls. Am J Clin Pathol 135: 146–156. 10.1309/AJCP9X8OZBEIQVVI 21173137

[19] Vona G, Sabile A, Louha M, Sitruk V, Romana S, et al (2000) Isolation by size of epithelial tumor cells : a new method for the immunomorphological and molecular characterization of circulatingtumor cells. Am J Pathol 156: 57–63. 10623654 10.1016/S0002-9440(10)64706-2 PMC1868645

[20] Adams D, Makarova O, Zhu P, Li S, Amstutz PT, et al.. (2011) Isolation of Circulating Tumor Cells by Size Exclusion using Lithography Fabricated Precision Microfilters [abstract]; April 2–6; Orlando, FL, USA. AACR.

[21] Lin HK, Zheng S, Williams AJ, Balic M, Groshen S, et al (2010) Portable filter-based microdevice for detection and characterization of circulating tumor cells. Clin Cancer Res 16: 5011–5018. 20876796 10.1158/1078-0432.CCR-10-1105 PMC2955786

[22] Xu T, Lu B, Tai YC, Goldkorn A (2010) A cancer detection platform which measures telomerase activity from live circulating tumor cells captured on a microfilter. Cancer Res 70: 6420–6426. 20663903 10.1158/0008-5472.CAN-10-0686 PMC2922429

[23] Zheng SY, Lin HK, Lu B, Williams A, Datar R, et al (2011) 3D microfilter device for viable circulating tumor cell (CTC) enrichment from blood. Biomed Microdevices 13: 203–213. 20978853 10.1007/s10544-010-9485-3 PMC3809998

[24] Tan SJ, Lakshmi RL, Chen P, Lim WT, Yobas L, et al (2010) Versatile label free biochip for the detection of circulating tumor cells from peripheral blood in cancer patients. Biosens Bioelectron 26: 1701–1705. 20719496 10.1016/j.bios.2010.07.054

[25] Gleghorn JP, Pratt ED, Denning D, Liu H, Bander NH, et al (2010) Capture of circulating tumor cells from whole blood of prostate cancer patients using geometrically enhanced differential immunocapture (GEDI) and a prostate-specific antibody. Lab Chip 10: 27–29. 20024046 10.1039/b917959c PMC3031459

[26] Nagrath S, Sequist LV, Maheswaran S, Bell DW, Irimia D, et al (2007) Isolation of rare circulating tumour cells in cancer patients by microchip technology. Nature 450: 1235–1239. 18097410 10.1038/nature06385 PMC3090667

[27] Stott SL, Hsu CH, Tsukrov DI, Yu M, Miyamoto DT, et al (2010) Isolation of circulating tumor cells using a microvortex-generating herringbone-chip. Proc Natl Acad Sci U S A 107: 18392–18397. 20930119 10.1073/pnas.1012539107 PMC2972993

[28] Dharmasiri U, Njoroge SK, Witek MA, Adebiyi MG, Kamande JW, et al (2011) High-throughput selection, enumeration, electrokinetic manipulation, and molecular profiling of low-abundance circulating tumor cells using a microfluidic system. Anal Chem 83: 2301–2309. 21319808 10.1021/ac103172y PMC4380022

[29] Stakenborg T, Liu C, Henry O, Borgen E, Laddach N, et al (2010) Automated genotyping of circulating tumor cells. Expert Rev Mol Diagn 10: 723–729. 20843197 10.1586/erm.10.66

[30] Talasaz AH, Powell AA, Huber DE, Berbee JG, Roh KH, et al (2009) Isolating highly enriched populations of circulating epithelial cells and other rare cells from blood using a magnetic sweeper device. Proc Natl Acad Sci U S A 106: 3970–3975. 19234122 10.1073/pnas.0813188106 PMC2645911

[31] Wang S, Liu K, Liu J, Yu ZT, Xu X, et al (2011) Highly efficient capture of circulating tumor cells by using nanostructured silicon substrates with integrated chaotic micromixers. Angew Chem Int Ed Engl 50: 3084–3088. 21374764 10.1002/anie.201005853 PMC3085082

[32] Coumans FAW, Doggen CJM, Attard G, de Bono JS, Terstappen LWMM (2010) All circulating EpCAM+CK+CD45− objects predict overall survival in castration-resistant prostate cancer. Ann Oncol 21: 1851–1857. 20147742 10.1093/annonc/mdq030

[33] Ligthart ST, Coumans FAW, Attard G, Mulick Cassidy A, de Bono JS, et al (2011) Unbiased and Automated Identification of a Circulating Tumour Cell Definition That Associates with Overall Survival. PLoS One 6: e27419. 22087312 10.1371/journal.pone.0027419 PMC3210171

[34] Coumans FAW, van Dalum G, Beck M, Terstappen L (in press) Filtration parameters influencing circulating tumor cell enrichment from whole blood. PLOS ONE. doi: 10.1371/journal.pone.0061774. 10.1371/journal.pone.0061774 PMC3637225 23658615

[35] Ligthart S, Bidard F-C, Decraene C, Bachelot T, Delaloge S, et al (2012) Unbiased quantitative assessment of Her-2 expression of circulating tumor cells in patients with metastatic and non-metastatic breast cancer. Ann Oncol. doi: 10.1093/annonc/mds625. 10.1093/annonc/mds625 23275633

[36] Tibbe AGJ, Miller MC, Terstappen LWMM (2007) Statistical considerations for enumeration of circulating tumor cells. Cytometry A 71A: 154–162. 10.1002/cyto.a.20369 17200956

[37] Kowalewska M, Chechlinska M, Markowicz S, Kober P, Nowak R (2006) The relevance of RT-PCR detection of disseminated tumour cells is hampered by the expression of markers regarded as tumour-specific in activated lymphocytes. Eur Journal Cancer 42: 2671–2674. 10.1016/j.ejca.2006.05.036 16978860

[38] Hou HW, Li QS, Lee GYH, Kumar AP, Ong CN, et al (2009) Deformability study of breast cancer cells using microfluidics. Biomed Microdevices 11: 557–564. 19082733 10.1007/s10544-008-9262-8

[39] Leong FY, Li Q, Lim CT, Chiam KH (2011) Modeling cell entry into a micro-channel. Biomech Model Mechanobiol 10: 755–766. 21104422 10.1007/s10237-010-0271-1

[40] Kan HC, Shyy W, Udaykumar HS, Vigneron P, Tran-Son-Tay R (1999) Effects of nucleus on leukocyte recovery. Ann Biomed Eng 27: 648–655. 10548334 10.1114/1.214

[41] Nibbering PH, Zomerdijk TPL, Corsel-Van Tilburg AJ, Van Furth R (1990) Mean cell volume of human blood leucocytes and resident and activated murine macrophages. J Immunol Methods 129: 143–145. 2110946 10.1016/0022-1759(90)90432-u

[42] Coumans FAW, Ligthart ST, Uhr JW, Terstappen LWMM (2012) Challenges in the Enumeration and Phenotyping of CTC. Clin Cancer Res 18: 5711–5718. 23014524 10.1158/1078-0432.CCR-12-1585

[43] Nguyen AT, Baggerman J, Paulusse JM, van Rijn CJ, Zuilhof H (2011) Stable Protein-Repellent Zwitterionic Polymer Brushes Grafted from Silicon Nitride. Langmuir 27: 2587–2594. 21291256 10.1021/la104657c

